# Safety Monitoring of JYNNEOS Vaccine During the 2022 Mpox Outbreak — United States, May 22–October 21, 2022

**DOI:** 10.15585/mmwr.mm7149a4

**Published:** 2022-12-09

**Authors:** Jonathan Duffy, Paige Marquez, Pedro Moro, Eric Weintraub, Yon Yu, Peter Boersma, James G. Donahue, Jason M. Glanz, Kristin Goddard, Simon J. Hambidge, Bruno Lewin, Ned Lewis, Douglas Rouse, Tom Shimabukuro

**Affiliations:** ^1^CDC Mpox Emergency Response Team; ^2^Marshfield Clinic Research Institute, Marshfield, Wisconsin; ^3^Kaiser Permanente Colorado Institute for Health Research, Denver, Colorado; ^4^Kaiser Permanente Vaccine Study Center, Kaiser Permanente Northern California, Oakland, California; ^5^Community Health Services, Denver Health, Denver, Colorado and Department of Pediatrics, University of Colorado School of Medicine, Denver, Colorado; ^6^Department of Research and Evaluation, Kaiser Permanente Southern California, Pasadena, California; ^7^Food and Drug Administration, Silver Spring, Maryland.

JYNNEOS (Modified Vaccinia Ankara vaccine, Bavarian Nordic) is recommended in the United States for persons exposed to or at high risk for exposure to *Monkeypox virus* during the 2022 monkeypox (mpox) outbreak (*1*). JYNNEOS is a live, nonreplicating viral vaccine licensed for the prevention of smallpox and mpox in adults aged ≥18 years, administered as a 0.5-mL 2-dose series given 28 days apart by subcutaneous injection (*2*). On August 9, 2022, the Food and Drug Administration (FDA) issued an Emergency Use Authorization (EUA) for administration of 0.1 mL doses by intradermal injection for adults aged ≥18 years as a strategy to increase vaccine supply, and administration of 0.5 mL doses subcutaneously for persons aged <18 years (*3*). During May 22–October 21, 2022, a total of 987,294 JYNNEOS vaccine doses were administered in the United States. CDC has monitored JYNNEOS vaccine safety using the Vaccine Adverse Event Reporting System (VAERS) and the Vaccine Safety Datalink (VSD) for vaccine recipients of all ages, and through single-patient emergency Investigational New Drug (EIND) procedures for persons aged <18 years vaccinated before August 9, 2022. The most common adverse health events reported to VAERS for adults were nonserious and included injection site reactions, which was consistent with the prelicensure studies. Adverse health events were reported at similar rates for doses received by intradermal and subcutaneous administration. Serious adverse events were rare in adults, and no serious adverse events have been identified among persons aged <18 years. Overall, postlicensure and postauthorization surveillance to date support JYNNEOS vaccine safety.

VAERS is a national passive surveillance system for adverse events after vaccination (*4*). VAERS accepts reports from health care providers, vaccine manufacturers, and the public. The JYNNEOS EUA requires reporting the following events to VAERS: vaccine administration errors (whether or not associated with an adverse event), serious adverse events (irrespective of attribution to vaccination), and cases of cardiac, thromboembolic, and neurovascular events.* Reported signs and symptoms are coded using Medical Dictionary for Regulatory Activities (MedDRA) terminology.^†^ Adverse events that are serious or of special interest are followed up by obtaining medical records, information from health care providers, and, in cases of death, death certificates and autopsy reports. Adverse events of special interest include anaphylaxis (an adverse event that can occur after any vaccine), and myocarditis, which is associated with older smallpox vaccines (*5*,*6*). Reports received and processed by October 21, 2022, were included.^§^ Adverse event reporting rates were calculated by dividing the number of reports by the number of vaccine doses administered during May 22–October 14 (to allow a minimum of 7 days for VAERS reporting) and reported to CDC by October 24, 2022 (*7*).

VSD is a collaboration between CDC and several integrated health care systems that uses electronic health record data to perform active vaccine safety surveillance (*8*). VSD identified medical visits with *International Classification of Diseases, Tenth Revision* diagnosis codes for myocarditis or pericarditis^¶^ that occurred within 30 days after either dose of JYNNEOS and verified the diagnosis using medical record review. Eight VSD health care systems contributed data to this assessment. For VAERS and VSD, rates and bivariate rate ratios (RRs) with associated 95% CIs were estimated and compared using Fisher’s exact test; analyses were conducted using OpenEpi software (version 3.01; OpenEpi).

CDC facilitated JYNNEOS EIND authorizations from FDA for 65 persons aged <18 years. CDC solicited information from vaccine providers about adverse events occurring during the 28 days after each dose. All activities described were reviewed by CDC and conducted consistent with applicable federal law and CDC policy.**

During the surveillance period (May 22–October 21, 2022), 987,294 JYNNEOS vaccine doses were administered in the United States, including 652,641 (66%) first doses and 334,568 (34%) second doses. Approximately one half (51%) of doses were administered intradermally, one third (34%) subcutaneously, and the remaining 15% by unknown or other routes. Overall, 90% of vaccinated persons were male. JYNNEOS vaccine was administered to 1,003 persons aged <18 years.

## Vaccine Adverse Event Reporting System

VAERS received 1,350 reports for JYNNEOS. Most reports were for males (84%), after dose 1 (63%), and for vaccine doses administered either intradermally (54%) or subcutaneously (25%) (Table 1). Approximately one half of reports (638; 47%) documented a vaccine administration error, 624 (98%) of which did not mention an adverse health event. The administration error reporting rate was higher for intradermal (818 per million doses administered) than for subcutaneous administration (314) (RR = 2.61; 95% CI = 2.10–3.26). The most common perceived vaccination error reported for intradermal administration was absence of a wheal without vaccine leakage on the first injection attempt (220 [54%] of 410 error reports). Among all VAERS reports, 685 (51%) documented an adverse health event. The reporting rates of adverse health events were similar for intradermal and subcutaneous administration (648 and 627 reports per million doses administered, respectively) (RR = 1.03; 95% CI = 0.87–1.24). The most common types of adverse health events reported differed by route of administration (Table 2).

**TABLE 1:** Characteristics of JYNNEOS vaccine recipients with reports submitted to the Vaccine Adverse Event Reporting System after vaccination (N = 1,350) — United States, May 22–October 21, 2022

Characteristic	No. (%)
**Sex**	
Male	1,134 (84)
Female	184 (14)
Not reported	32 (2)
**Age group, yrs**	
0–17	13 (1)
18–49	1,013 (75)
50–64	247 (18)
≥65	68 (5)
Not reported	9 (1)
**Dose in series**	
First	850 (63)
Second	317 (23)
Not reported or other	183 (14)
**Route of administration**	
Intradermal	732 (54)
Subcutaneous	334 (25)
Intramuscular	155 (11)
Not reported or other	129 (10)
**JYNNEOS administered with other vaccines the same day**	
Yes	33 (2)
No	1,317 (98)
Seriousness classification*	
Nonserious	1,336 (99)
Serious	14 (1)
**JYNNEOS vaccine administration error reported**	
Yes	638 (47)
No	712 (53)
**Adverse health event reported**	
Yes	685 (51)
No	665 (49)

* Based on the Code of Federal Regulations, classification of a serious adverse event includes a report of one of the following: death, life-threatening illness, hospitalization or prolongation of hospitalization, permanent disability, congenital anomaly, or birth defect.

**TABLE 2:** Reporting rates for the 10 most frequently reported adverse health events* after JYNNEOS vaccine receipt, by route of administration^†^ — Vaccine Adverse Event Reporting System, United States, May 22–October 21, 2022

Route of administration/Health event	No. of reports	Reporting rate^§^ (95% CI)
**Intradermal (n = 325)**		
Injection site erythema	75	150 (118–188)
Dizziness	66	132 (102–168)
Urticaria	60	120 (91–154)
Injection site swelling	51	102 (76–134)
Syncope	43	86 (62–116)
Erythema	42	84 (60–113)
Loss of consciousness	41	82 (59–111)
Injection site pruritus	40	80 (57–109)
Hyperhidrosis	38	76 (54–104)
Pruritus	33	66 (45–92)
**Subcutaneous (n = 212)**		
Injection site erythema	36	107 (75–148)
Injection site swelling	36	107 (75–148)
Injection site pain	34	101 (70–141)
Pain	29	86 (57–123)
Erythema	28	83 (55–120)
Dizziness	27	80 (53–116)
Headache	26	77 (50–113)
Fatigue	25	74 (48–109)
Injection site pruritus	23	68 (43–102)
Pyrexia	23	68 (43–102)

* Excluding vaccination errors and deviations from recommendations.

^†^ Licensed and authorized routes of administration only.

^§^ Reports per million doses administered; total number of intradermal doses administered = 501,228 and subcutaneous doses administered = 337,950.

Fourteen reports (1%) were classified as serious. Two deaths in males aged 37 and 58 years were reported, both within 2 days of vaccination. In one case, drowning was the cause of death. The death certificate is pending for the other case. Nine reports were classified as serious because of hospitalization for the following events: myocarditis (two), pericarditis (two), appendicitis (one), aseptic meningitis (one), atrial fibrillation (one), idiopathic thrombocytopenic purpura (one), and methemoglobinemia (one). Three vaccinated persons reported the following events as representing disability or permanent damage in their own assessment: injection site discoloration (one), injection site pain (one), and injection site scar (one).

The myocarditis reporting rate was 1.53 cases per million doses within 30 days after receipt of dose 1 and 2.99 after dose 2. Three reports of anaphylaxis within 24 hours of vaccination were received (overall reporting rate = 3.04; 95% CI = 0.63–8.88 cases per million doses administered) (Table 3).

**TABLE 3:** Reporting rates for adverse events of special interest after JYNNEOS vaccine receipt — Vaccine Adverse Event Reporting System, United States, May 22–October 21, 2022

Adverse event/Dose	Postvaccination risk interval	No. of reports	No. of doses administered	Reporting rate* (95% CI)
Myocarditis				
After dose 1	30 days	1	652,641	1.53 (0.04–8.54)
After dose 2		1	334,568	2.99 (0.08–16.65)
**Anaphylaxis**				
After dose 1	24 hours	2	652,641	3.06 (0.37–11.07)
After dose 2		1	334,568	2.99 (0.08–16.65)

* Reports per million doses administered.

VAERS received 13 reports for persons aged <18 years, one of which included an adverse health event (syncope). The other reports for persons in this age group were related to vaccine administration errors, most commonly inadvertent intradermal rather than subcutaneous administration (six), which is the authorized route of administration for persons aged <18 years.

## Vaccine Safety Datalink

As of October 21, 2022, a total of 43,253 JYNNEOS doses had been administered to persons in the VSD population, representing approximately 4.3% of all doses administered nationally. Among 25,659 males and 1,953 females who received dose 1, 58% and 37%, respectively, also received dose 2. One case of myocarditis was identified after each dose in males. The incidence among males after dose 1 was 39 per million doses (95% CI = 0.1–217.1) and after dose 2 was 67 (95% CI = 1.7–374.4).

## Emergency Investigational New Drug Authorizations

Among the 65 persons aged <18 years for whom CDC obtained EIND authorization for vaccination, 55 were confirmed to have received ≥1 vaccine dose. CDC also received vaccine follow-up information for seven additional persons aged <18 years who were vaccinated under the EUA. Overall, vaccine recipients ranged in age from 4 months to 17 years, and 58% were male. Information about whether adverse events occurred was received for 57 of the 62 persons aged <18 years vaccinated. Adverse events were reported for 10 (18%) of 57 after the first dose and five (21%) of 24 after the second dose. Most were injection site reactions, including pain, erythema, swelling, and induration. Systemic adverse events included fever, fatigue, and headache. No serious adverse events were reported.

## Discussion

Monitoring of JYNNEOS vaccine safety in the United States during the 2022 mpox outbreak has not identified any new or unexpected safety concerns among adults or persons aged <18 years. The VAERS reporting rate of anaphylaxis after JYNNEOS is similar to rates previously published after receipt of other vaccines (*9*). JYNNEOS safety in persons aged <18 years had not been assessed before this outbreak. Pediatric vaccine safety information collected to date has not identified any concerning adverse events.

Not all adverse events that occur after vaccination are caused by the vaccine. Currently, no evidence indicates that either of the two deaths reported to VAERS after JYNNEOS administration were caused by the vaccine. These two deaths within 2 days of vaccination are less than the number expected to occur by chance alone. For example, during 2019, an average of six deaths occurred daily per 1 million men aged 35–39 years (*10*).

Myocarditis is associated with live, replicating smallpox vaccines, such as ACAM2000, with incidence point estimates for symptomatic cases ranging from 78 to 5,230 cases per million persons within 30 days after vaccination (*5*,*6*). The background myocarditis rate has been estimated to be 21.6 cases per million in a 30-day period (*5*). The VAERS myocarditis reporting rate (1.53 and 2.99 per million first and second vaccine doses administered, respectively) is at least seven times lower than the background rate. VSD myocarditis incidence estimates have wide CIs that encompass both the background rate and the lower incidence estimates for the replicating smallpox vaccines. Current data do not suggest an increased risk for myocarditis after receipt of JYNNEOS, but the possibility of a small risk cannot be excluded.

Vaccine administration errors have been reported more often following intradermal than subcutaneous administration of JYNNEOS vaccine. The most common issue reported has been a wheal not forming with the initial injection. CDC’s interim clinical considerations for use of JYNNEOS state that absence of a wheal without vaccine leakage may be counted as valid administration (*1*). 

The findings in this report are subject to at least three limitations. First, VAERS is a passive reporting system and is subject to underreporting and reporting biases; for example, the two myocarditis cases identified by VSD were not reported to VAERS. Common, nonserious adverse events, such as injection site reactions, are less likely to be reported compared with serious adverse events. Second, comparison of VAERS reporting rates to published background rates might not signal a potential risk if the published rate is higher than the vaccinated population’s true background rate. Finally, VSD might not receive JYNNEOS vaccine administration data for all out-of-network doses; vaccination history might be recorded more often for patients with a medical visit for an adverse event, which could lead to overestimating adverse event incidence.

JYNNEOS postlicensure and postauthorization vaccine safety surveillance findings to date are consistent with those observed in the clinical trials, and support JYNNEOS vaccine safety with no new or unexpected safety concerns identified. Serious adverse events were rare among adults, and none have been identified among persons aged <18 years. CDC and FDA will continue to monitor the safety of JYNNEOS. Health care providers should continue to report adverse events after JYNNEOS to VAERS.^††^

**Summary**

**What is already known about this topic?**

JYNNEOS vaccine has been used in a real-world setting for the first time during the 2022 monkeypox (mpox) outbreak, including intradermal administration under a Food and Drug Administration (FDA) Emergency Use Authorization.

**What is added by this report?**

During May 22–October 21, 2022, nearly 1 million JYNNEOS doses were administered in the United States. The vaccine safety profile was consistent with prelicensure studies. The most common adverse health events reported were nonserious and included injection site reactions. Serious adverse events were rare among adults, and no serious adverse events have been identified among persons aged <18 years.

**What are the implications for public health practice?**

Surveillance supports JYNNEOS vaccine safety. CDC and FDA will continue to monitor the safety of JYNNEOS.

## Footnotes

* The JYNNEOS EUA requires health care providers and the vaccine manufacturer to report serious adverse events (irrespective of attribution to vaccination), and cases of cardiac, thromboembolic, and neurovascular events. Health care providers are also required to report vaccine administration errors (whether or not associated with an adverse event). Based on the Code of Federal Regulations, a serious adverse event is defined as occurring if one of the following is reported: death, life-threatening illness, hospitalization or prolongation of hospitalization, permanent disability, congenital anomaly, or birth defect.

† A single VAERS report might be assigned more than one MedDRA preferred term; not all terms are medically confirmed diagnoses. https://www.meddra.org/how-to-use/basics/hierarchy

§ Processed VAERS reports are those that have been MedDRA-coded, deduplicated, and have undergone quality assurance and quality control.

¶ Myocarditis and pericarditis *International Classification of Diseases, Tenth Revision* codes included B33.22*, B33.23*, I30.*, I31.9*, I40.*, and I51.4. An asterisk indicates that any trailing digit values were included.

** 45 C.F.R. part 46, 21 C.F.R. part 56; 42 U.S.C. Sect. 241(d); 5 U.S.C. Sect. 552a; 44 U.S.C. Sect. 3501 et seq.

††
https://vaers.hhs.gov
